# Novel Tetrahydro-[1,2,4]triazolo[3,4-*a*]isoquinoline Chalcones Suppress Breast Carcinoma through Cell Cycle Arrests and Apoptosis

**DOI:** 10.3390/molecules28083338

**Published:** 2023-04-10

**Authors:** Mahmoud I. M. Darwish, Ahmed M. Moustafa, Asmaa M. Youssef, Mohamed Mansour, Ahmed I. Yousef, Abdelfatteh El Omri, Hossam H. Shawki, Magda F. Mohamed, Hamdi M. Hassaneen, Ismail A. Abdelhamid, Hisashi Oishi

**Affiliations:** 1 Department of Biochemistry, Faculty of Veterinary Medicine, Zagazig University, Zagazig 44511, Egypt; 2 Department of Comparative and Experimental Medicine, Nagoya City University Graduate School of Medical Sciences, Nagoya 467-8601, Japan; 3 Zoology Department, Faculty of Science, Al-Azhar University, Cairo 11884, Egypt; 4 Animal Health Research Institute, Agriculture Research Center, Giza 12619, Egypt; 5 National Gene Bank of Egypt, Giza 12916, Egypt; 6 Molecular Physiology Division, Faculty of Science, Beni-Suef University, Beni-Suef 62511, Egypt; 7 Surgical Research Section, Department of Surgery, Hamad Medical Corporation, Doha 3050, Qatar; 8 Department of Chemistry, Faculty of Science, Cairo University, Giza 12613, Egypt

**Keywords:** breast cancer, chalcones, methoxy group effect, MDA, Luc4T1, cell cycle, cytotoxicity, docking, cIAP1

## Abstract

Chalcones are interesting anticancer drug candidates which have attracted much interest due to their unique structure and their extensive biological activity. Various functional modifications in chalcones have been reported, along with their pharmacological properties. In the current study, novel chalcone derivatives with the chemical base of tetrahydro-[1,2,4]triazolo[3,4-a]isoquinolin-3-yl)-3-arylprop-2-en-1-one were synthesized, and the structure of their molecules was confirmed through NMR spectroscopy. The antitumor activity of these newly synthesized chalcone derivatives was tested on mouse (Luc-4T1) and human (MDA-MB-231) breast cancer cell lines. The antiproliferative effect was evaluated through SRB screening and the MTT assay after 48 h of treatment at different concentrations. Interestingly, among the tested chalcone derivatives, chalcone analogues with a methoxy group were found to have significant anticancer activity and displayed gradient-dependent inhibition against breast cancer cell proliferation. The anticancer properties of these unique analogues were examined further by cytometric analysis of the cell cycle, quantitative PCR, and the caspases-Glo 3/7 assay. Chalcone methoxy derivatives showed the capability of cell cycle arrest and increased *Bax*/*Bcl2* mRNA ratios as well as caspases 3/7 activity. The molecular docking analysis suggests that these chalcone methoxy derivatives may inhibit anti-apoptotic proteins, particularly cIAP1, BCL2, and EGFRK proteins. In conclusion, our findings confirm that chalcone methoxy derivatives could be considered to be potent drug candidates against breast cancer.

## 1. Introduction

Breast cancer is the most prevalent type of cancer in women worldwide, with a total of 2.26 million cases each year, 11.7% of all cancer cases, and 24.5% of the cancer cases in women [1]. Additionally, it accounts for 15.5% of annual cancer deaths among women [1]. The use of cytotoxic chemotherapeutic drugs is the currently established treatment option for breast cancer, either alone or in combination with other medical procedures such as surgery and radiotherapy. A number of FDA-approved chemotherapeutic drugs such as 5-fluorouracil (**5-FU**), capecitabine, docetaxel, doxorubicin, epirubicin, gemcitabine, methotrexate, paclitaxel, tamoxifen citrate, and nucleosides [2] are available as therapeutic options for cancer. However, they are still considered toxic agents and are associated with long-term adverse effects [2,3].

A continuous research effort is ongoing to develop potent new therapeutics or to improve chemotherapeutics. Modern oncology aims to develop novel natural or synthetic compounds with antitumor properties. Such potential compounds are chalcones, which belong to a class group of flavonoids [4]. Chalcones typical structure is a central 1,3-diphenyl-prop-2-en-1-one structure known as a chalcone core, which consists of two aromatic rings jointed by the three-carbon α, β-unsaturated carbonyl system [5]. Chalcones (1-(2′-hydroxyphenyl)-3-phenylprop-2-en-1-ones) and their derivatives are often obtained from natural sources such as citrus fruits, vegetables, and spices [6,7] or as a result of chemical synthesis [8,9]. Both natural and synthetic analogs are reported to exhibit various biological properties including anticancer, anti-inflammatory, and antioxidant activities [10,11,12,13].

Recently, chalcones have been shown to induce apoptosis and cell cycle arrest in various cancer cells, including breast cancer [14,15]. Inhibitory effects of some chalcones have been linked to the tumor suppressor p53 [16]. The functional groups of synthetic chalcone derivatives might influence such bioactivity depending on their position on the aryl rings (A and B). The chalcones exist in two isomers: trans (*E*) and cis (*Z*). The *E* isomer is the most stable and hence the most prevalent structure of the chalcones [17]. Trimethoxy chalcone exhibits anticancer activity in a variety of human cancer cell lines [18], making it an attractive scaffold for studying its anticancer activity.

Motivated by the above-mentioned facts and in continuation of our interest in the synthesis of bioactive heterocycles [19,20,21,22,23,24,25,26,27,28], in the present study, we synthesized a new series of chalcone derivatives. The cytotoxic, antiproliferative, and proapoptotic effects of newly synthesized compounds were analyzed. The anticancer activity of new chalcone analogues with a methoxy group tested on mouse and human breast cancer cell lines showed a greater significant anticancer potency than the parent compound. Compounds **3a** and **5a** exhibited a strong suppression of cell growth in association with cell cycle arrests and apoptosis.

## 2. Results

### 2.1. Synthesis of the Chalcones

**Figure 1:**
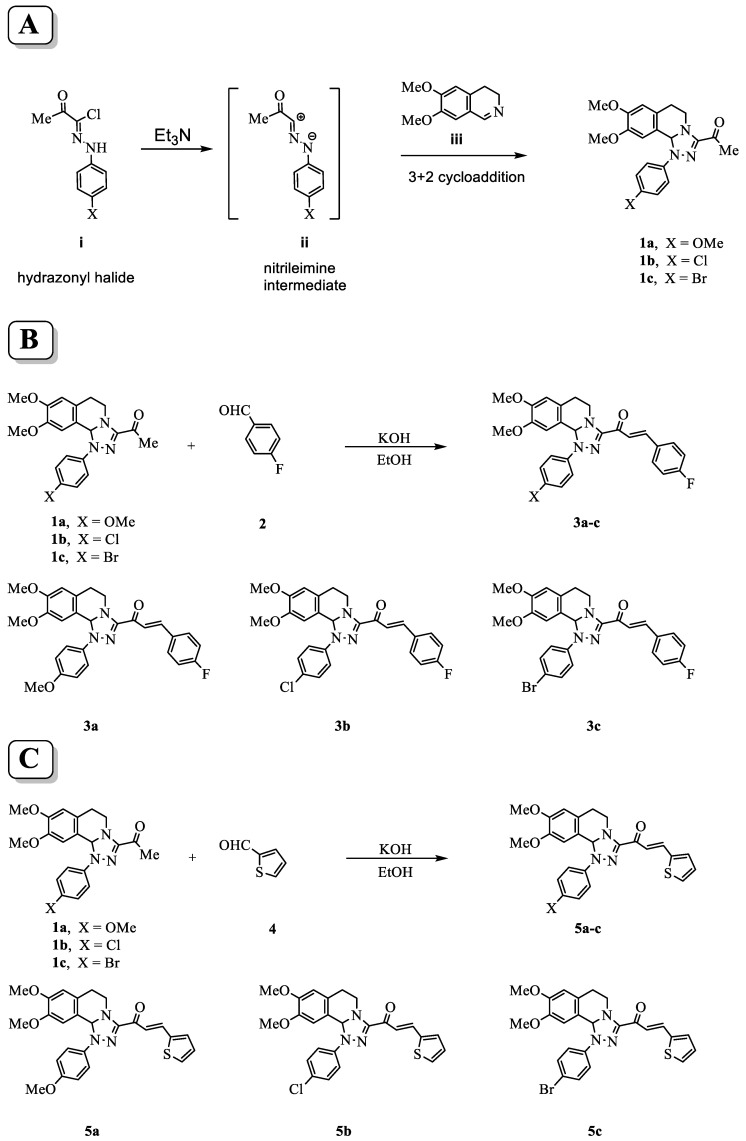
Scheme for the synthesis of tetrahydro-[1,2,4]triazolo[3,4-*a*]isoquinolin-3-yl)-3-arylprop-2-en-1-one chalcone derivatives. (**A**) Procedures of the reaction to form the parent compounds (**1a**–**c**) through the reactions of nitrilimine **ii** with 3,4-dihydro-6,7-dimethoxyisoquinoline **iii**. (**B**) Formation of the chalcone derivatives (**3a**–**c**) by Claisen–Schmidt condensation of compounds (**1a–c**) with 4-fuorobenzaldehyde **2**. (**C**) Formation of the (**5a–c**) by Claisen–Schmidt condensation of compound (**1a**–**c**) with thiophene-2-carbaldehyde **4**.

A high yield and purity of starting 3-acetyl-8,9-dimethoxy-1-(4-methoxyphenyl)-1,5,6,10b-tetrahydro-[1,2,4]triazolo[3,4-*a*]isoquinoline **1a**, 3-acetyl-8,9-dimethoxy-1-(4-chlorophenyl)-1,5,6,10b-tetrahydro-[1,2,4]triazolo[3,4-*a*]isoquinoline **1b**, and 3-acetyl-8,9-dimethoxy-1-(4-bromophenyl)-1,5,6,10b-tetrahydro-[1,2,4]triazolo[3,4-*a*]isoquinoline **1c**, were synthesized using the described procedure in the scheme at (Figure 1A) through the reactions of nitrilimine **ii** with 3,4-dihydro-6,7-dimethoxyisoquinoline **iii**. Then, Claisen–Schmidt condensation of compound (**1a**–**c**) with equimolar amounts of substituted aldehydes **2** and **4** in the presence of potassium hydroxide solution leads to the formation of the corresponding chalcone derivatives (**3a**–**c**) shown in scheme Figure 1B and (**5a**–**c**) shown in scheme Figure 1C. The structures of the formed products were elucidated by inspection of their spectral data.

### 2.2. In Vitro Cytotoxicity Screening of Chalcone-Based Compounds

The in vitro cytotoxic screening was performed against the six newly synthesized chalcone compounds **3a**, **3b**, **3c**, **5a**, **5b**, and **5c**, using the SRB assay. The mouse breast cancer cells Luc-4T1 and the human breast cancer cells MDA-MB-231 were treated with or without each compound independently for 48 h at a final concentration of 20 µg/mL. In order to evaluate the drug’s activity, we used **5-FU**, an approved chemotherapeutic drug, as a positive control. Results indicated that out of six tested compounds, five showed a lower viability percentage than the **5-FU** in both mouse and human cell lines (Table 1). The cell viability percentages of Luc-4T1 were 52.5 ± 11.9, 64.3 ± 15.6, 57.6 ± 13.2, 79.6 ± 9.3, 92.1 ± 2.1, 81.6 ± 4.3, and 88.5 ± 0.6 for **3a**, **3b**, **3c**, **5a**, **5b**, **5c**, and **5-FU**, respectively. The cell viability percentages of MDA-MB-231 were 49.7 ± 12.7, 30.9 ± 8.3, 40.6 ± 6.8, 39.4 ± 12.5, 31.7 ± 11.5, 25.9 ± 3.3, and 64 ± 11.6 for **3a**, **3b**, **3c**, **5a**, **5b**, **5c**, and **5-FU**, respectively. These data indicated that screening of the chalcone compounds **3a**, **3b**, **3c**, **5a**, and **5c** except **5b** showed a better cytotoxic effect compared to **5-FU.**

**Figure 2:**
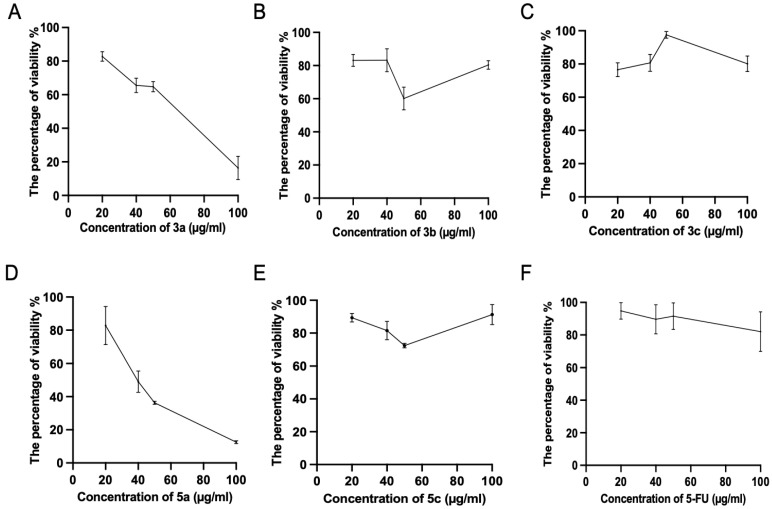
Cytotoxic evaluations of the tested chalcone compounds against the Luc-4T1 cell line. The cell line was treated for 48 h with different concentrations (20, 40, 50, and 100 μg/mL) of the individual chalcones: (**A**) compound **3a**, (**B**) compound **3b**, (**C**) compound **3c**, (**D**) compound **5a**, (**E**) compound **5c**, and (**F**) **5-FU**, and the percentage of viability was determined through MTT assay. The values are means ± SD (*n* = 4), and two independent experiments were examined.

**Figure 3:**
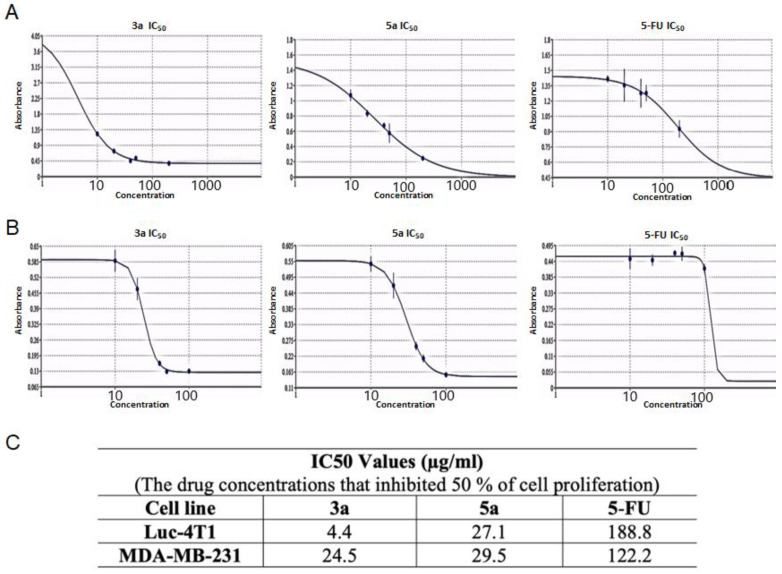
The IC50 values of the chalcone methoxy derivatives 3a and 5a. IC50 was determined through the MTT assay by non-linear regression using (**A**) Mouse Luc-4t1 and (**B**) Human MDA-MB-231 cell lines after 48 h of treatment, with **5-FU** as a positive control against. (**C**) Summary of the IC50 values. The Quest Graph™ IC50 Calculator [29] was used to calculate the IC50.

Subsequently, we tested the five compounds with different doses to see the dose-dependent effect with the increasing of concentration (Figure 2). Cytotoxic evaluations of the chalcone derivatives and **5-FU** as a positive control were performed against the Luc-4T1 cell line at different concentrations (20, 40, 50, and 100 μg/mL). After a 48 h treatment with different concentrations under the same experimental conditions, compounds **3a** and **5a** showed a gradual cytotoxic effect with increasing concentrations, but not the other tested compounds **3b**, **3c**, **5b**, and **5c**. In contrast, the positive control **5-FU** showed a slight gradual cytotoxic effect, but its level of cytotoxicity was much lower than that of compounds **3a** and **5a**. Therefore, to ascertain these data and calculate the IC50, we repeated the experiment with a wider range of concentrations between (10-200 μg/mL) for the two promising compounds (**3a** and **5a**) as well as the positive control **5-FU** on the cell lines from the two origins of mouse and human (Figure 3). As the same as before, both compounds showed better cytotoxic effects with lower IC50s against the two cell lines when compared to **5-FU**. The IC50 on Luc4T1 was 4.4, 27.1, and 188.8 μg/mL for compounds **3a**, **5a**, and **5-FU**, respectively (Figure 3A,C). In addition, the IC50 on MDA-MB-231 was 24.5, 29.5, and 122.2 μg/mL for compounds **3a**, **5a**, and **5-FU**, respectively (Figure 3B,C). These data indicated that the introduction of a methyl group at position 4 of the phenyl group (compounds **3a** and **5a**) significantly improved the growth-inhibitory activity of breast carcinoma.

### 2.3. Chalcone Analogues with the Methoxy Group Induced Cell Cycle Arrests and Apoptosis in Luc4T1 Cells

**Figure 4:**
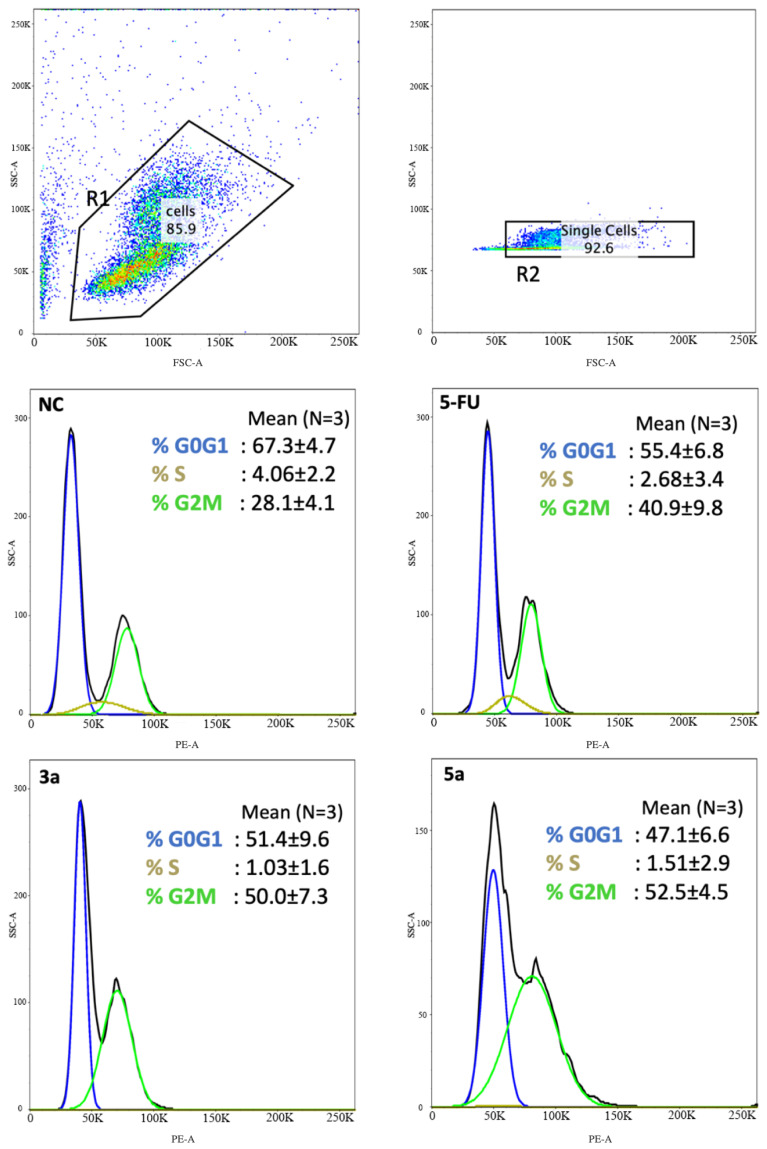
Chalcone methoxy derivatives induce cell cycle arrest in Luc-4T1 cells. Cells were treated for 24 h with 20 μg/mL of compound **3a**, compound **3c**, **5-FU** as a positive control, or untreated as controls (NC). The cells were then harvested, fixed in 70% ethanol, and stained with PI for FACS analysis. The percentage of cells in each phase of the cell cycle was shown as mean ± SD over the histograms. Two independent experiments were performed. NC; non-treated cells.

The compounds with the strongest activity in the MTT test (compounds **3a** and **5a**) were selected for further research on the nature of cell death. Anti-proliferation is an important mechanism for therapeutic agents to suppress tumor growth. The effects of the compounds **3a** and **5a** on the Luc-4T1 cell cycle progression were examined after 24 h of treatment with a concentration around the IC50 (20 μg/mL). FACS profiles indicated that compound **3a** induced cell cycle arrest at the G2/M phase (50.0 ± 7.3%) as well as compound **5a** (52.5 ± 4.5%) in comparison to the non-treated cells (28.1 ± 4.1%), while the positive control **5-FU** induced cell cycle arrest at G2/M phase lower than the tested chalcone derivatives (40.9 ± 9.8%) (Figure 4). The cell cycle distribution of the non-treated cells (G0G1 = 67.3 ± 4.7%, S = 4.06 ± 2.2%, G2/M = 28.1 ± 4.1%), **5-FU** (G0G1 = 55.4 ± 6.8%, S = 2.68 ± 3.4%, G2/M = 40.9 ± 9.8%), compound **3a** (G0G1 = 51.4 ± 9.6%, S = 1.03 ± 1.6%, G2/M = 50.0 ± 7.3%), and compound **5a** (G0G1 = 47.1 ± 6.6%, S = 1.51 ± 2.9%, G2M = 52.5 ± 4.5%). The transition from one phase to another phase in the cell cycle is a very important point for controlling cell proliferation. These data suggest that compounds **3a** and **5a** cause mitotic arrest, as shown by the accumulation of cells in the G2/M phase (Figure 4).

**Figure 5:**
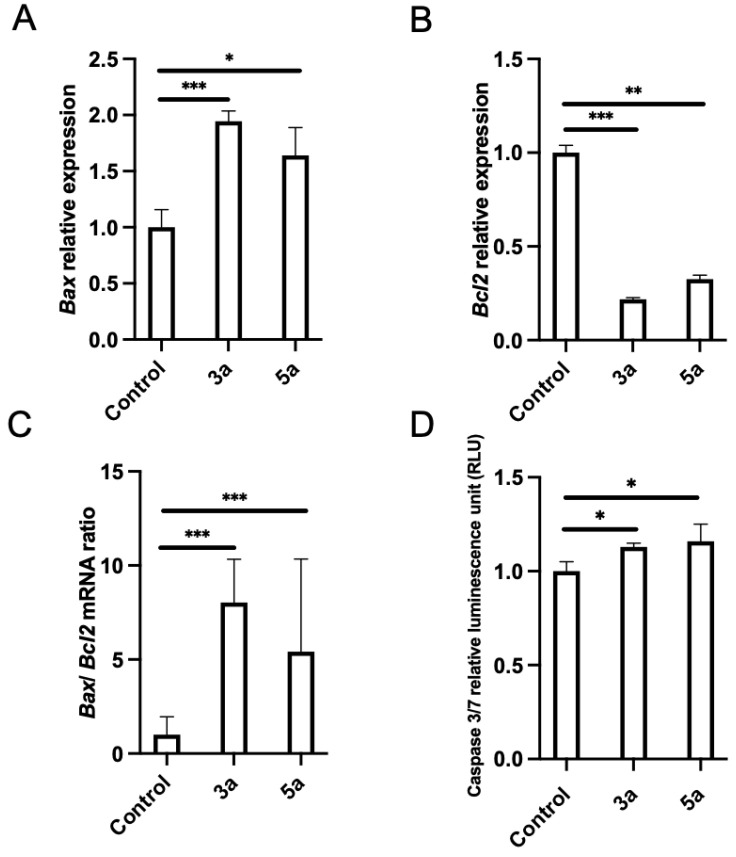
Effect of chalcone methoxy derivatives on the levels of apoptotic markers in Luc-4T1 cells. The cells were cultured with 10 µg/mL of compound **3a**, compound **5a**, or a control vehicle for 24h, and then either RNA extraction or the Caspase-Glo 3/7 assay was performed. The relative transcription levels of (**A**) *Bax*, (**B**) *Bcl2*, and (**C**) *Bax*/*Bcl2* mRNA ratio were determined. Gene expression levels were normalized to *Hprt*. (**D**) Caspace 3/7 relative luminescence. Bars represent the mean ± SD of four replicates from two independent experiments. * *p* < 0.05, ** *p* < 0.01, and *** *p* < 0.001 represent a statistical difference.

Pro-apoptosis is another important mechanism for therapeutic agents to eliminate malignant cells. The analysis for apoptotic key elements was performed using quantitative PCR. *Bax* and *Bcl2* are two important regulator genes in the mitochondrial apoptotic pathway. The *Bcl2* gene product is thought to contribute to oncogenesis by suppressing signals that induce apoptotic cell death. We checked the effects of chalcone methoxy derivatives (**3a** and **5a**) on *Bax* and *Bcl2* expression. Luc-4T1 cells were treated with a slightly lower concentration (10 μg/mL) of **3a**, **5a**, or the vehicle, for 24 h to monitor molecular changes before the cells entered cell cycle arrest or died due to cytotoxicity. The mRNA expression level of the proapoptotic gene *Bax* was found to be significantly up-regulated when treated with compound **3a** (*p* < 0.001) and compound **5a** (*p* < 0.05) relative to the control (Figure 5A), while the expression of the anti-apoptotic gene *Bcl2* was significantly down-regulated when treated with compound **3a** (*p* < 0.001) and compound **5a** (*p* < 0.01) relative to the control (Figure 5B). Because the experimental evidence suggests that the balance between anti-apoptotic and pro-apoptotic members of the *Bcl2* family is a much better determinant of the sensitivity to apoptosis, we evaluated the *Bax/Bcl2* expression ratio. The ratio of *Bax/Bcl2* was significantly increased (Figure 5C). Thus, up-regulation of the *Bax*/*Bcl2* expression ratio by the two compounds **3a** and **5a** relative to the control promoted the apoptotic death of the Luc-4T1 cell line. Concentrations of active caspase-3/7, the main enzymes involved in the process of apoptosis, were measured to confirm the apoptotic cascade (Figure 5D). Luc-4T1 cells were treated with the same concentration (10 μg/mL) of **3a**, **5a**, or the vehicle for 24 h. By using the Caspase-Glo 3/7 assay kit, we measured Caspase 3/7 activity. Our results showed a significant increase in relative luminescence (*p* < 0.05) in the case of both compounds **3a** and **5a** compared with the control vehicle (Figure 5D). These data suggested that both compounds **3a** and **5a** strongly stimulated apoptotic induction of Luc-4T1. Taken together, the data above indicated that compounds **3a** and **5a** could induce apoptosis and cell cycle arrest at the G2/M phase in Luc-4T1 breast cancer cells.

### 2.4. Modeling Simulation Analysis of Chalcone Analogues with the Methoxy Group

Compounds **3a** and **5a** were found to inhibit the expression of the anti-apoptotic gene *Bcl2*. To gain insight into their potential mode of action, we performed molecular docking studies to investigate the binding modes of these promising compounds against targeted anti-apoptotic proteins. This was important because inhibiting these proteins is a crucial strategy for reducing cancer growth. The targeted proteins were epidermal growth factor receptor tyrosine kinase (EGFRTK), cyclin-dependent kinase 2 (CDK2), cellular inhibitor of apoptosis protein 1 (cIAP1), mouse double minute 2 (MDM2), and B-cell lymphoma 2 (BCL2). The PDB IDs of the targeted proteins were 1m17, 2c6o, 4kmn, 4wt2, and 2w3l, respectively. Table 2 and Table 3 showed different binding energy readings of the co-crystallized standard ligand in comparison to the energy readings of the tested compounds **3a** and **5a**. The two tested compounds **3a** and **5a** proved a strong binding affinity toward the targeted anti-apoptotic protein c-IAP1 with binding energies (S) −22.12 and −24.08 Kcal/mol, respectively, in comparison to their co-crystallized ligand −14.40 Kcal/mol. Moreover, compound **5a** ensured affinity (−20.07 Kcal/mol) much more than standard ligand (−18.25 Kcal/mol) regarding the anti-apoptotic BCL2 protein, while compound **3a** exhibited almost the same binding affinity against that protein with binding energy (−18.09 Kcal/mol). On the other hand, compounds **3a** and **5a** proposed comparable binding activities toward the EGFRTK protein with binding energies of −23.60 and −22.56 Kcal/mol, respectively, with respect to the standard ligand of −23.91 Kcal/mol. Regarding the CDK2 active domain, the two compounds showed moderate effects relative to the standard ligand. In contrast, the two compounds **3a** and **5a** illustrated similar and weak binding affinities of −24.72 and −24.60 Kcal/mol toward MDM2 protein considering the standard ligand −41.29 Kcal/mol.

**Figure 6:**
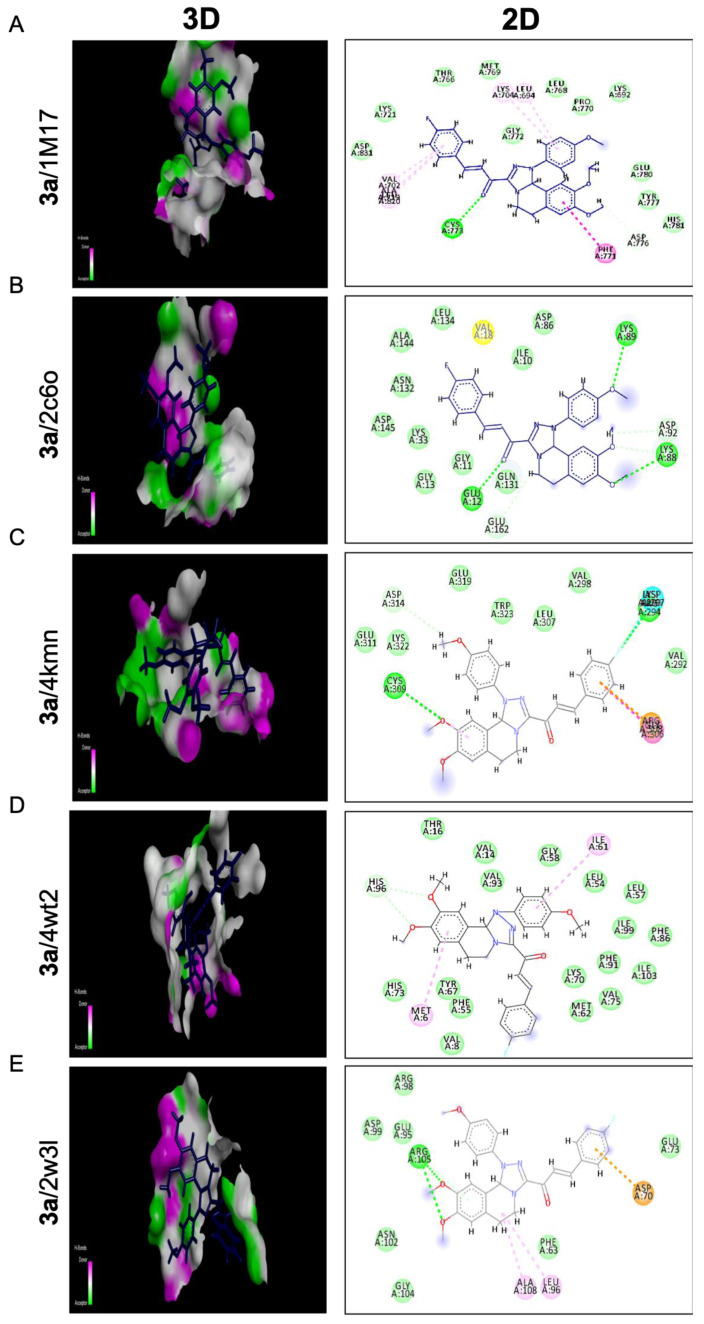
Molecular docking analysis of chalcone compound 3a. Representation of 3D and 2D modeling into the active site of (**A**) epidermal growth factor receptor tyrosine kinase domain (1m17), (**B**) cyclin-dependent kinase 2 domain (2c6o), (**C**) cellular inhibitor of apoptosis protein 1 domain (4kmn), (**D**) mouse double minute 2 domain (4wt2), and (**E**) B-cell lymphoma 2 domain (2w3l).

Compound **3a** exposed the best affinity and selectivity toward the active site 4kmn of the anti-apoptotic protein (cIAP1). As illustrated in Figure 6C, compound **3a** fitted well and blocked active site 4kmn through seven different types of interactions (conventional H bonds, halogen (fluorine), Pi-cation, Amid-Pi stacked, Pi-alkyl, Van der Waals, and carbon-hydrogen bond). Compound **3a** blocked the active site of EGFRTK with a binding energy of −23.60 Kcal/mol comparable to that of a standard ligand −23.91 Kcal/mol. As exposed in Figure 6A, compound **3a** exerted its action through five different interactions as follows: conventional H bonds, Amid-Pi stacked, Pi-alkyl, Van der Waals, and carbon-hydrogen bond. Moreover, compound **3a** confirmed compatible activity against the BCL2 domain −18.09 Kcal/mol relative to the standard ligand −18.25 Kcal/mol with six interactions as illustrated in Figure 6E. Interactions achieved by **3a** with the active site of 2w3l were conventional H bonds, Alkyl, Pi-alkyl, Van der Waals, Pi-anion, and Pi-donor H bonds. On the other hand, compound **3a** showed a moderate effect toward cdk2 protein with a binding energy −21.54 Kcal/mol regarding the standard ligand −26.73 Kcal/mol. However, it showed good binding affinity toward the 2c6o domain, as illustrated in Figure 6B. The compound demonstrated different types of interactions toward that domain such as conventional H bonds, carbon-hydrogen bonds, Pi-alkyl, Van der Waals, and Amide-Pi stacked. Regarding the 4wt2 domain, compound **3a** was weakly fitted into that active site with a binding affinity of −24.72 Kcal/mol compared to the standard ligand −41.29 Kcal/mol. As seen in (Figure 6D), interactions considered in that active canter by compound **3a** were only C-H bond, Pi-Alkyl, and Van der Waals.

**Figure 7:**
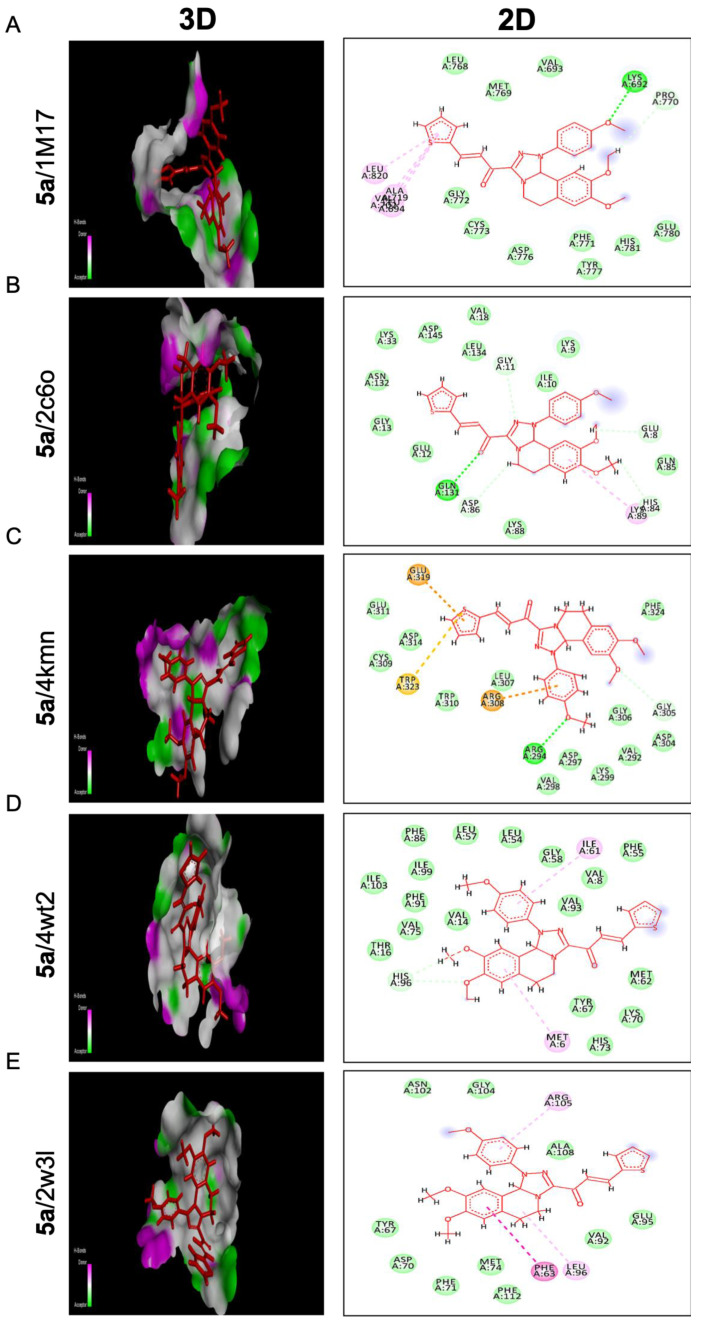
Molecular docking analysis of chalcone compound 5a. Representation of 3D and 2D modeling into the active site of (**A**) epidermal growth factor receptor tyrosine kinase domain (1m17), (**B**) cyclin-dependent kinase 2 domain (2c6o), (**C**) cellular inhibitor of apoptosis protein 1 domain (4kmn), (**D**) mouse double minute 2 domain (4wt2), and (**E**) B-cell lymphoma 2 domain (2w3l).

Referring to compound **5a**, it represents the best binding activities toward two proteins; cIAP1 and BCL2 with binding energies of −24.08 and −20.07 Kcal/mol relative to the co- crystallized ligands of −14.40 and −18.25 Kcal/mol, respectively. Seven different bindings toward the 4kmn domain were shown (Figure 7C) as follows: conventional H bonds, carbon-hydrogen bond, Pi-alkyl, Van der Waals, Pi-sulfur, Pi-Cation, and Pi-Anion. Moreover, compound **5a** was strongly fitted into the 2w3l domain through four interactions (Pi-Pi T shaped, Pi-Alkyl, Alkyl, and Van der Waals) as outlined in Figure 7E. With respect to the 1m17 domain, compound **5a** offered comparable binding affinity against that domain with the energy binding of −22.56 Kcal/mol as the same as the standard ligand −23.91 Kcal/mol. Figure 7A revealed that compound **5a** was interacting with the active site of the EGFRTK domain within different interactions such as conventional H bonds, carbon-hydrogen bonds, Pi-alkyl, and Van der Waals. On the other hand, compound **5a** indicated moderate binding affinity toward CDK2 protein with binding energy of −20.47 Kcal/mol compared to the standard ligand −26.73 Kcal/mol and by blotting **5a** versus the 2C6O domain through several interactions: conventional H bonds, carbon-hydrogen bond, Pi-alkyl, and Van der Waals (Figure 7B). In addition, compound **5a** showed a weak binding affinity toward the MDM2 protein with binding energy of −24.60 Kcal/mol in comparison to the standard ligand −41.29 Kcal/mol. Compound **5a** was bonded through only Pi-Alkyl and Van der Waals bonds as shown (Figure 7D). This proposed that fluorobenzene and thiophene moieties were responsible for all the above-mentioned interactions, especially those involving the anti-apoptotic proteins cIAP1, BCL2, and EGFRK, which regulate metastasis and the cell cycle.

## 3. Discussion

An investigation has been conducted on the potential therapeutic effects of novel chalcone derivatives on breast carcinoma. Breast cancer is a major health problem throughout the world. Current therapeutic options are not fully effective as a result of the complexity of the disease and the long-term adverse effects [3,30]. Chalcones are a class of flavonoids that have shown promise in various bioactivities, including anticancer properties [10,11,12,13]. Therefore, the modification was made to synthesize chalcone analogues containing a methoxy, chloro, or bromine group at position 4 of the phenyl group and to see how such a modification affects the antitumor activity in vitro. In the current study, we first screened the antiproliferative activity of the newly synthesized compounds (**3a**–**c** and **5a**–**c**) in breast cancer cell lines originating from mouse (Luc-4T1) and human (MDA-MB-231). As shown in Table 1 and Figure 2 and Figure 3, using SRB and MTT assays, the authors found that two out of the six tested compounds, **3a** and **5a**, displayed dose-dependent inhibition effects with a lower IC50 than the positive control **5-FU** against both cell lines. Interestingly, the two most active derivatives were chalcones with methoxy analogues, indicating that the introduction of a methyl group at position 4 of the phenyl group significantly improved their growth-inhibitory activity against breast carcinoma. In accordance with our finding, a recent study by Pawlak et al. demonstrated that the introduction of a methoxy functional group further improved chalcone’s cytotoxic potency against canine lymphoma or leukemia [18], although their strongest antitumor activity is shown by compounds containing a methoxy group in position 2 or 3 more than position 4 of the B ring. It has been demonstrated that methoxylated flavonoids easily penetrate the cells [31], resulting in better bioavailability and absorption. Moreover, the prolonged metabolism of methoxylated flavonoids might contribute to their promising cytotoxic activity. In this connection, further analysis of the chalcone with methoxy analogs (compounds **3a** and **5a**) was found to induce cell cycle arrest at the G2/M phase and induce apoptosis (Figure 4 and Figure 5). Cell cycle progression is a crucial process for controlling cancer cell proliferation. Current results showed that compounds **3a** and **5a** induced G2/M phase cell cycle arrest in breast cancer cells, which may have contributed to their anti-proliferative activity. This finding matches with previous studies showing that chalcone derivatives can induce cell cycle arrest at different phases in various cancer cell lines [13,15,32]. The specific mechanism by which compounds **3a** and **5a** induce cell cycle arrest in the G2/M phase remains to be explained, but it may involve the disruption of microtubule dynamics or the activation of checkpoint pathways. Apoptosis is another critical process for eliminating malignant cells, and the induction of apoptosis is an important mechanism of action for anticancer therapeutic agents. Our study reported that compounds **3a** and **5a** induced apoptosis in breast cancer cells, as evidenced by an increased *Bax*/*Bcl2* expression ratio and the increase in active caspase-3/7, confirming the apoptotic cascade. These results suggest that chalcone with methoxy analogues may promote apoptosis in breast cancer cells through the mitochondrial apoptotic pathway.

The precise mechanisms of chalcone activating proapoptotic and anti-proliferative pathways need more investigation, however; we investigated the molecular binding modes of two compounds (**3a** and **5a**) against five targeted anti-apoptotic proteins that play a central role in fighting cancer. Molecular docking investigations were conducted to evaluate the compounds’ binding activities against the epidermal growth factor receptor tyrosine kinase (EGFRTK), cyclin-dependent kinase 2 (CDK2), cellular inhibitor of apoptosis protein 1 (cIAP1), mouse double minute 2 (MDM2), and B-cell lymphoma 2 (BCL2), as illustrated in Table 2 and Table 3, Figure 6 and Figure 7. The binding energies of the compounds were compared to the binding energies of co-crystallized standard ligands. The results showed that compound **3a** had the best affinity and selectivity toward the active site 4kmn of the anti-apoptotic protein (cIAP1). It also showed compatible activity against the EGFRTK and BCL2 domains. Moreover, compound **5a** displayed the best binding activities toward two proteins: cIAP1 and BCL2. All these binding modes confirmed our suggestion that the methoxy group played an important role in the enhanced binding activity of compounds **3a** and **5a** into the active site of the target proteins and hence inhibit cancer progression.

The proapoptotic activity of chalcones is still a frequently discussed subject of scientific research. There are numerous scientific reports that describe the different mechanisms of proapoptotic actions of compounds from this group [33,34,35]. In our study, we primarily focused on the structure-function of chalcones containing an alteration at position 4 of the phenyl group with methoxy, chloro, or bromine on breast cancer cell death and, secondarily, investigated their possible anticancer mechanisms of action. To the best of our knowledge, the current study is the first to demonstrate that newly synthesized chalcone analogues containing a methoxy group over a chloro or bromine group could potentially be an effective treatment option for breast cancer. Further studies are needed to elucidate the molecular mechanisms and to evaluate their efficacy and safety in preclinical and clinical trials.

## 4. Materials and Methods

### 4.1. Chemistry and Structure Elucidation

A Stuart melting point device was used to measure each compound’s melting points and they were uncorrected. DMSO-*d*_6_ was used as a solvent to record the ^1^H and ^13^C (the attached proton test, APT) NMR spectra at 300 MHz and 75 MHz, respectively, on a Varian Gemini NMR spectrometer using TMS as an internal standard. Chemical shifts are reported in *δ* units (ppm). The IR spectra were recorded as KBr using a Bruker-vector 22 spectrophotometer FTIR. A Shimadzu GMSS -QP-1000 EX mass spectrometer was used to measure the mass spectra at 70 eV. All the elemental analyses were performed at the Microanalytical Center, Cairo University (Giza, Egypt).

### 4.2. Synthesis of Chalcones (***3a–c***) and (***5a–c***) and NMR Analysis

A mixture of [1,2,4]triazolo[3,4-*a*]isoquinolin-3-yl)ethan-1-one **1** (0.351 g, 1 mmol) and appropriate aldehydes **2** or **4** (1 mmol) was dissolved in 20 mL ethanol. KOH (20%, 5 mL) was added to the mixture at 0–5 °C. The reaction mixture was stirred at room temperature for 5 h, then poured over ice containing HCl. The yellow solid obtained was then filtered and washed with water. After precipitation, the yellow solid was separated using a vacuum pump, then dried at a constant temperature (80–90 °C) in an oven. The crude product was crystallized in a proper solvent to yield chalcones.

*(E)-1-(8,9-Dimethoxy-1-(4-methoxyphenyl)-1,5,6,10b-tetrahydro-[1,2,4]triazolo[3,4-a]isoquinolin-3-yl)-3-(4-fluorophenyl)prop-2-en-1-one* (**3a**). Yield: (78%) as a pale-yellow solid (from acetonitrile); m.p 138–140 °C. IR (KBr, cm^−1^): 1665 (CO); ^1^H NMR (400 MHz, DMSO-*d*_6_): δ, ppm: 2.6–2.8 (m, 2H, H6), 3.4 (s, 3H, OMe), 3.5–3.6 (m, 1H, H5), 3.7 (s, 3H, OMe), 3.8 (s, 3H, OMe), 4.3–4.4 (m, 1H, H5), 6.6 (s, 1H, H10b), 6.7 (s, 1H, H7), 6.9 (s, 1H, H10), 7.0–7.9 (m, 10H, 2 vinyl-H + Ar-H); ^13^C NMR (100 MHz, DMSO-*d*_6_): δ, ppm: 27.3, 41.9, 55.7, 55.8, 55.9, 79.5, 109.8, 112.5, 115.1, 116.6, 118.4, 123.0, 126.7, 129.1, 131.4, 131.7, 137.0, 140.0, 147.3, 148.9, 149.5, 155.2, 162.5, 164.9, 178.8; MS (EI): *m/z* = 487 (M^+^). Anal. Calcd. for C_28_H_26_FN_3_O_4_ (487.53): C, 68.98; H, 5.38; N, 8.62. Found: C, 69.12; H, 5.51; N, 8.83.
*(E)-1-(1-(4-Chlorophenyl)-8,9-dimethoxy-1,5,6,10b-tetrahydro-[1,2,4]triazolo[3,4-a]isoquinolin-3-yl)-3-(4-fluorophenyl)prop-2-en-1-one* (**3b**). Yield: (85%) as a pale-yellow solid (from dioxane); m.p 178–180 °C. IR (KBr, cm^−1^): 1668 (CO); ^1^H NMR (400 MHz, DMSO-*d*_6_): δ, ppm: 2.7–2.9 (m, 2H, H6), 3.5 (s, 3H, OMe), 3.6–3.7 (m, 1H, H5), 3.7 (s, 3H, OMe), 4.1–4.2 (m, 1H, H5), 6.6 (s, 1H, H10b), 6.8 (s, 1H, H7), 6.9 (s, 1H, H10), 7.3–7.9 (m, 10H, 2 vinyl-H + Ar-H); ^13^C NMR (100 MHz, DMSO-*d*_6_): δ, ppm: 27.4, 41.9, 55.8, 56.0, 77.8, 109.1, 112.5, 116.5, 116.6, 122.7, 124.9, 127.4, 128.8, 129.6, 131.6, 141.1, 142.7, 147.6, 149.1, 150.2, 156.5, 162.7, 165.1, 179.5; MS (EI): *m/z* = 491 (M^+^). Anal. Calcd. for C_27_H_23_ClFN_3_O_3_ (491.95): C, 65.92; H, 4.71; N, 8.54. Found: C, 66.15; H, 4.56; N, 8.76.
*(E)-1-(1-(4-Bromophenyl)-8,9-dimethoxy-1,5,6,10b-tetrahydro-[1,2,4]triazolo[3,4-a]isoquinolin-3-yl)-3-(4-fluorophenyl)prop-2-en-1-one* (**3c**). Yield: (88%) as a pale-yellow solid (from dioxane); m.p 174–176 °C. IR (KBr, cm^−1^): 1666 (CO); ^1^H NMR (400 MHz, DMSO-*d*_6_): δ, ppm: 2.7–2.8 (m, 2H, H6), 3.5 (s, 3H, OMe), 3.7 (s, 3H, OMe), 3.9–3.9 (m, 1H, H5), 4.1–4.2 (m, 1H, H5), 6.6 (s, 1H, H10b), 6.8 (s, 1H, H7), 6.9 (s, 1H, H10), 7.3–7.9 (m, 10H, 2 vinyl-H + Ar-H); ^13^C NMR (100 MHz, DMSO-*d*_6_): δ, ppm: 27.4, 41.9, 55.8, 56.0, 66.8, 77.7, 109.1, 112.5, 112.6, 116.4, 116.7, 116.8, 127.4, 128.8, 131.6, 132.4, 141.1, 143.1, 147.6, 149.1, 150.2, 162.7, 165.2, 179.5; MS (EI): *m/z* = 536 (M^+^). Anal. Calcd. for C_27_H_23_BrFN_3_O_3_ (536.40): C, 60.46; H, 4.32; N, 7.83. Found: C, 60.61; H, 4.57; N, 7.97.
*(E)-1-(8,9-Dimethoxy-1-(4-methoxyphenyl)-1,5,6,10b-tetrahydro-[1,2,4]triazolo [3,4-a]isoquinolin-3-yl)-3-(thiophen-2-yl)prop-2-en-1-one* (**5a**). Yield: (80%) as a red solid (from acetonitrile); m.p 168–170 °C. IR (KBr, cm^−1^): 1670 (CO); ^1^H NMR (300 MHz, DMSO-*d*_6_): δ, ppm: 2.6–2.8 (m, 2H, H6), 3.4 (s, 3H, OMe), 3.7 (s, 3H, OMe), 3.7–3.7 (m, 1H, H5), 4.2–4.3 (m, 1H, H5), 6.6 (s, 1H, H10b), 6.7 (s, 1H, H7), 6.9 (s, 1H, H10), 7.0–7.00 (m, 2H, Ar-H and vinyl-H), 7.2–7.2 (dd, 1H, thiophene-H, *J* = 3.6, 5.0 Hz), 7.3–7.4 (m, 3H, Ar-H), 7.6 (d, 1H, thiophene-H, *J* = 3.6 Hz), 7.7 (d, 1H, thiophene-H, *J* = 5.0 Hz), 7.80 (d, 1H, vinyl-H, *J* = 15.6 Hz);^13^C NMR (75 MHz, DMSO-*d*_6_): δ, ppm: 27.3, 41.9, 55.7, 55.8, 55.9, 79.6, 109.7, 112.4, 115.2, 118.3, 121.5, 126.6, 129.1, 129.3, 130.4, 133.3, 134.1, 137.1, 140.2, 147.3, 148.9, 149.6, 155.3, 178.4; MS (EI): *m/z* = 483 (M^+^). Anal. Calcd. for C_26_H_25_N_3_O_4_S (475.56): C, 65.67; H, 5.30; N, 8.84. Found: C, 65.79; H, 5.47; N, 8.98.
*(E)-1-(1-(4-Chlorophenyl)-8,9-dimethoxy-1,5,6,10b-tetrahydro-[1,2,4]triazolo[3,4-a]isoquinolin-3-yl)-3-(thiophen-2-yl)prop-2-en-1-one* (**5b**). Yield: (84%) as a red solid (from acetonitrile); m.p 178–180 °C. IR (KBr, cm^−1^): 1671 (CO); ^1^H NMR (300 MHz, DMSO-*d*_6_): δ, ppm: 2.7–2.9 (m, 2H, H6), 3.5 (s, 3H, OMe), 3.6–3.7 (m, 1H, H5), 3.7 (s, 3H, OMe), 4.1–4.2 (m, 1H, H5), 6.6 (s, 1H, H10b), 6.8 (s, 1H, H7), 6.9 (s, 1H, H10), 7.2–7.2 (m, 1H, thiophene-H), 7.3–7.4 (m, 5H, vinyl-H), 7.6 (d, 1H, thiophene-H, *J* = 3.6 Hz), 7.8 (d, 1H, thiophene-H, *J* = 5.0 Hz), 7.9 (d, 1H, vinyl-H, *J* = 15.7 Hz);^13^C NMR (75 MHz, DMSO-*d*_6_): δ, ppm: 27.3, 41.9, 55.8, 56.1, 77.8, 109.1, 112.5, 116.4, 121.2, 124.8, 127.4, 128.8, 129.4, 129.7, 130.9, 133.8, 135.2, 140.0, 142.7 147.6, 149.1, 150.3, 178.9; MS (EI): *m/z* = 479 (M^+^). Anal. Calcd. for C_25_H_22_ClN_3_O_3_S (479.98): C, 62.56; H, 4.62; N, 8.75. Found: C, 62.41; H, 4.81; N, 8.95.
(*E)-1-(1-(4-Bromophenyl)-8,9-dimethoxy-1,5,6,10b-tetrahydro-[1,2,4]triazolo[3,4-a]isoquinolin-3-yl)-3-(thiophen-2-yl)prop-2-en-1-one* (**5c**). Yield: (78%) as a red solid (from acetonitrile); m.p 182–184 °C. IR (KBr, cm^−1^): 1668 (CO); ^1^H NMR (300 MHz, DMSO-*d*_6_): δ, ppm: 2.7–2.8 (m, 2H, H6), 3.5 (s, 3H, OMe), 3.9 (s, 3H, OMe), 4.1–4.2 (m, 2H, H5), 6.6 (s, 1H, H10b), 6.8 (s, 1H, H7), 6.9 (s, 1H, H10), 7.2–7.9 (m, 8H, Ar + thiophene-H + vinyl-H), 7.9 (d, 1H, vinyl-H, *J* = 15.7 Hz);^13^C NMR (75 MHz, DMSO-*d*_6_): δ, ppm: 27.4, 41.9, 55.8, 55.8, 56.0, 77.7, 109.0, 112.5, 112.6, 116.7, 121.2, 128.8, 129.4, 131.0, 132.5, 133.8, 135.2, 140.0, 143.1 147.6, 149.1, 150.3, 179.0; MS (EI): *m/z* = 524 (M^+^). Anal. Calcd. for C_25_H_22_BrN_3_O_3_S (524.43): C, 57.26; H, 4.23; N, 8.01. Found: C, 57.42; H, 4.41; N, 8.13.

### 4.3. Cell Culture

Experimental work and the handling of cell lines were performed according to Nagoya City University guidelines. Mouse Luc-4T1 was obtained from the JCRB Cell Bank (Accession number #JCRB1447) and human MDA-MB-231 was obtained from the ATCC (Accession number #HTB-26). Both are breast cancer cells that were maintained as a monolayer culture in RPMI-1640 (Sigma, St. Louis, MO, USA) supplemented with L-glutamine, sodium pyruvate, non-essential amino acids, 1% penicillin-streptomycin, and 10% fetal bovine serum (Gibco BRL, Grand Island, NY, USA). Cells were grown at 37 °C in a humidified atmosphere containing 5% CO_2_.

### 4.4. SRB Assay

The SRB assay is used for the toxicity screening of compounds on adherent cells in a 96-well plate. The cells were cultured with the tested compounds, or **5-FU** (FUJIFILM Wako Pure Chemical Corporation, Osaka, Japan), for 48 h at a final concentration of 20 µg/mL. After the incubation period, cells were fixed with ice-cold absolute ethanol for 1 h at −20 °C, followed by staining with 0.4% (*w/vol*) sulforhodamine b (code) for 30 min in the dark at RT. Cells were repeatedly washed to remove the excess dye with 1% (*v/v*) acetic acid. The protein-bound dye was resolved with a 10 mM unbuffered Tris solution for optical density determination at 565 nm using a microplate reader. Cell viability was expressed as a percentage of the control values.

### 4.5. MTT Assay

Cell viability was estimated by a colorimetric 3-(4,5-dimethylthiazol-2-y1)-2,5-diphenyl-tetrazolium bromide (MTT) assay. Briefly, cells were seeded for 24 h at 1 × 10^4^ cells/well in 96-well plates, followed by treatment with different doses of the tested compounds or **5-FU** for another 24 h. The culture medium was then replaced with fresh serum-free medium containing MTT solution (MTT cell proliferation assay kit, Nacalai tesque, No. 23506-80) and incubated for 3 h at 37 °C. The formed formazan crystals were solubilized by MTT solvent on a shaker and the absorbance was measured at 590 nm on a microplate reader (Spectra MAX 340, Molecular Devices Co., Sunnyvale, CA, USA). Cells were treated with a medium containing 0.1% DMSO as a vehicle and survival was expressed as a percentage of the absorbance relative to that of control cells.

### 4.6. Cell Cycle Analysis

The cell cycle was analyzed using FACS Canto II (BD Biosciences, San Jose, CA, USA). Briefly, cells were seeded for 24 h at 1 × 10^5^ cells/well in a 12-well plate, followed by treatment with 20 µg/mL of the tested compounds, **5-FU**, or vehicle for 24 h. Thereafter, the medium was removed, and the cells were harvested by trypsinization, washed with PBS, and fixed with 70% ethanol for 30 min at 4 °C. The cells were then washed twice with PBS and stained with propidium iodide (50 μg/mL) and RNase A (100 μg/mL) in PBS at room temperature for 1 h. The stained cells were immediately analyzed on the flow cytometer for relative DNA content. The resulting data were analyzed using FlowJo software v10.8.2 to determine cell cycle distribution.

### 4.7. Quantitative PCR Analysis

Total RNA was extracted from Mouse Luc-4T1 cells after 24 h of treatment using ISOGEN II (311-07361, NIPPON GENE, Tokyo, Japan), and QuantiTect Reverse Transcription Kit (QIAGEN, 205313) was used to synthesize cDNA, each according to the manufacturer’s instructions. The quantitative PCR reaction was carried out on the QuantStudio 12K Flex Real-Time PCR System (Thermo Fisher Scientific, Waltham, MA, USA) with PowerUp™ SYBR^®^-Green Master Mix (A25776, Thermo Fisher Scientific). The following primers were used: forward 5′-TTAGAGAGATGCGAGGAACCG-3′ and reverse 5′-GGGACAAGTAAACCTGGAAGAA-3′ for *Bcl2*; 5′-TGCAGAGGATGATTGCTGAC-3′ and reverse 5′-GATCAGCTCGGGCACTTTAG-3′ for *Bax*; and forward 5′-TTGTTGTTGGATATGCCCTTGACTA-3′ and reverse 5′-AGGCAGATGGCCACAGGACTA-3′ for *Hprt*. The results were expressed in Cycle threshold (Ct). Relative quantitation was assessed according to the calculation of delta-delta Ct. All qPCR analyses were performed in duplicate.

### 4.8. Caspase-Glo 3/7 Assay

Caspase-3/7 activity was measured using the Caspase-Glo^®^ 3/7 assay kit (Promega Corporation, Madison, WI, USA, 8090) according to the manufacturer’s protocol. In brief, cells were seeded for 24 h at 1 × 10^4^ cells/well in a white-walled 96-well plate, followed by treatment with 10 µg/mL of the tested compounds for another 24 h. The Caspase-Glo^®^ 3/7 reagents were brought to room temperature for 30 min, added to the culture cells, and then incubated for 3 h at RT. The luminescence was measured using a SpectraMax Microplate Luminometer (Molecular Devices, CA, USA).

### 4.9. Molecular Docking Analysis

The molecular docking studies were performed using the Molecular Operating Environment (MOE) version 2009.10 program. The target compound**s** were drawn with the MOE builder interface and subjected to local energy minimization using the included MOPAC. Then, the resulting structure was subjected to global energy minimization via Systematic Conformational Search where the RMS gradient and RMS distance were set to be 0.01 kcal/mole and 0.1A°, respectively. After the energy calculation of our compound was achieved, the lowest value of energy was chosen for docking with epidermal growth factor receptor kinase (EGFRK), cyclin-dependent kinase 2, (CDK2), and Mouse double minute 2 homolog (MDM2) proteins. The X-ray crystallographic structure of the proteins was downloaded from the protein data bank (PDB ID: 1m17, 2C6O, and 4wt2, respectively). Before the molecular simulation step, the proteins were prepared as follows: Firstly, protonation of the target protein with its standard ligand. Then, unwanted co-ligands and water chains were removed from the protein. After that, the active site was selected by the MOE alpha site finder. Finally, the prepared proteins were docked with the target compound and the results were compared with the self-docking results where the proteins were docked with their co-crystallized ligands ([6,7-Bis(2-methoxy-ethoxy)quinazoline-4-yl]-(3-ethynylphenyl)amine), (O6-cyclohexylmethoxy-2-(4′-sulphamoylanilino)purine), and (4-({[(3R,5R,6S)-1-[(1S)-2-(tert-butylsulfonyl)-1-cyclopropylethyl]-6-(4-chloro-3fluorophenyl)-5-(3-chlorophenyl)-3-methyl-2-oxopiperidin-3-yl]acetyl}amino)-2-methoxybenzoic acid). The final results were saved as pdb format files, which were then visualized through BIOVIA Discovery Studio V6.1.0.15350 in 2D and 3D forms.

### 4.10. Statistical Analysis

All results are representative of at least two independent experiments. The data were presented as mean ± standard deviation (SD) and statistical comparisons between two groups were determined by a two-tailed Student’s *t*-test, while for comparing more than one group, a one-way ANOVA was used. A probability value of <0.05 was considered significant.

## 5. Conclusions

In the present study, novel chalcone derivatives were synthesized and evaluated for their antitumor activity against cancer cells of two origins (mouse and human) breast carcinoma. Results showed that chalcone with a methoxy group exhibited enhanced cytotoxicity, cell cycle arrest, and proapoptotic activity, leading to reduced cell growth. Their inhibitory effect on anti-apoptotic proteins, particularly cIAP1, BCL2, and EGFRK proteins was also confirmed by molecular docking.

## Figures and Tables

**Table 1:** SRB screening for six chalcone compounds on mouse and human breast cancer cell lines.

Compound	Cell Viability (%)	
	Luc-4T1	MDA-MB-231
**3a**	52.5 ± 11.9	49.7 ± 12.7
**3b**	64.3 ± 15.6	30.9 ± 8.3
**3c**	57.6 ± 13.2	40.6 ± 6.8
**5a**	79.6 ± 9.2	39.4 ± 12.5
**5b**	92.1 ± 2.1	31.7 ± 11.5
**5c**	81.6 ± 4.3	25.9 ± 3.3
**5-FU**	88.5 ± 0.6	64.0 ± 11.6

**Table 2:** Standard ligand energy readings against (1m17, 2c6o, 4kmn, 4wt2, and 2w31) domains.

	1m17 (EGFRTK)	2c6o (CDK2)	4kmn (cIAP1)	4wt2 (MDM2)	2w3l (BCL2)
**S**	−23.91	−26.73	−14.40	−41.29	−18.25
**RMSD**	1.15	2.29	5.00	1.06	4.61
**E-Place**	−57.22	−90.37	−77.58	−79.71	−44.35
**E-Score**	−10.29	−11.27	−9.38	−11.88	−9.72

S: represent Gibbs free energy, RMSD: represent root mean squared deviation, and E: represent energy.

**Table 3:** Tested compounds **3a** and **5a** energy readings against (1m17, 2c6o, 4kmn, 4wt2, and 2w31) domains.

	1m17 (EGFRTK)	2c6o (CDK2)	4kmn (cIAP1)	4wt2 (MDM2)	2w3l (BCL2)
	**3a**				
**S**	−23.60	−21.54	−22.12	−24.72	−18.09
**RMSD**	2.16	2.244	1.21	1.64	2.13
**E-Place**	−44.22	−69.75	−79.61	−53.25	−43.34
**E-Score**	−8.80	−9.64	−7.65	−8.87	−7.77
	**5a**				
**S**	−22.56	−20.47	−24.08	−24.60	−20.07
**RMSD**	1.94	3.22	1.97	1.79	1.25
**E-Place**	−67.01	−58.25	−65.40	−75.19	−78.05
**E-Score**	−8.79	−9.06	−9.95	−9.22	−8.96

S: represent Gibbs free energy, RMSD: represent root mean squared deviation, and E: represent energy.

## Data Availability

All data used to support the findings of this study are included within the article.

## Sample Availability

Samples of the compounds (**3a**–**c**) and (**5a**–**c**) are available from the authors.
